# The Third Joint Meeting on Lung Cancer of the FHU OncoAge (University Côte d’Azur, Nice, France) and the University of Texas MD Anderson Cancer Center (Houston, TX, USA). Understanding New Therapeutic Options and Promising Predictive Biomarkers for Lung Cancer Patients

**DOI:** 10.3390/cancers14174327

**Published:** 2022-09-04

**Authors:** Paul Hofman, George A. Calin, Sandurai A. Mani, Christophe Bontoux, Marius Ilié, Ignacio I. Wistuba

**Affiliations:** 1 Laboratory of Clinical and Experimental Pathology, Pasteur Hospital, Université Côte d’Azur, 06000 Nice, France; 2 Biobank-Related Hospital (BB-0033-00025), Pasteur Hospital, 06000 Nice, France; 3 FHU OncoAge, Pasteur Hospital, Université Côte d’Azur, 06000 Nice, France; 4 Inserm U1081, CNRS UMR 7413, IRCAN, 06100 Nice, France; 5 Department of Translational Molecular Pathology, The University of Texas MD Anderson Cancer Center, Houston, TX 77030, USA

We are proud and happy to present this Special Issue, a follow-up to the third joint meeting on lung cancer of the FHU OncoAge (University Côte d’Azur, Nice, France) and the University of Texas MD Anderson Cancer Center (Houston, TX, USA), which was held virtually on 4 October 2021. This meeting was devoted to better understanding new mechanisms of lung carcinogenesis and novel therapeutic options for lung cancer patients. Moreover, some promising biomarkers for non-small cell lung carcinoma (NSCLC) were also discussed.

In this Special Issue, we have brought together several articles focusing on recent topics related to lung cancer in the fields of fundamental, translational, and clinical research [1,2,3,4,5,6,7,8,9,10,11,12]. Basic research studies are essential to improve our knowledge of lung cancer carcinogenesis. Therefore, discoveries from these basic studies can help to develop future therapeutics or new diagnostics, prognostics, or predictive biomarkers [5,8,11,12]. In particular, tissue and circulating biomarkers from the coding and non-coding regions of the genome that are predictive of the response or resistance to different immunotherapies or targeted therapies are currently being evaluated or used in clinical trials or, more recently, in daily practice [1,2,10,13,14,15,16,17]. Certain biomarkers are also being tested but may be envisaged as optimizing approaches to precision medicine for NSCLC patients [5,7,18].

Novel therapeutic algorithms have been introduced for early and resectable stages of NSCLC. They include targeted and immune therapies with or without chemotherapy for neo and/or adjuvant treatments [19,20]. The development of some of these treatments is ongoing in clinical trials, while osimertinib can now be used in daily clinical practice as an adjuvant treatment with or without chemotherapy if a deletion in exon 19 of *EGFR* or if the L858 mutation in *EGFR* is detected on tumor tissue [19]. The results of some clinical trials require validation via studies on larger numbers of patients, but these studies are nonetheless promising and provide hope that new treatments will be introduced in clinical practice soon [19,21]. For example, new therapies will certainly target the *KRAS* mutation of NSCLC, in particular G12C *KRAS,* and will be used as first- or second-line treatment [1].

Several studies in translational research have improved knowledge of the resistance mechanisms that emerge in patients on targeted therapies, which explains the tumor progression and relapse of the patient [2]. Recently, identified genomic alterations can also explain the phenomena of primitive resistance to targeted treatments [22]. A better understanding of these different mechanisms should allow optimal care for patients with NSCLC and the introduction of new adapted and targeted treatments [2].

The introduction of several technological approaches associated with the discovery of novel diagnostic and predictive biomarkers for targeted and immune therapies has made the management of tissue and cytological samples increasingly complex in thoracic oncology. In fact, more than 80% of the samples obtained from patients with lung cancer are small in size (e.g., tissue, transthoracic or bronchial biopsies, cytological samples in particular of bronchial, lymph node or pleural origin). In this regard, and with the knowledge that it is essential to manage samples in an optimal way, significant research has been conducted surrounding the notion of “small samples” advocated by the WHO in their 2021 classification of lung cancers [23]. The increase in the number of biomarkers to be rapidly identified in NSCLC patients in daily practice requires the use of next-generation sequencing techniques [4]. An integrative approach will undoubtedly become necessary in the future, associating molecular genetic analyses with tumor tissue and/or fluids (from blood or other origins), multiplex immunochemical analyses, and algorithms based on artificial intelligence [3,4,24,25,26].

Despite the different therapeutic advances in the framework of care for lung cancer patients, studies focusing on the early detection of these cancers still need to be considered through the development of liquid biopsies and circulating biomarkers [27,28,29,30,31]. Along with early detection of lung cancer, we must also focus on preventive measures and identifying populations at risk of developing lung cancer, which could help to quickly reduce the number of deaths from lung cancers [9,29,32].
